# Children and young people’s participation in developing interventions in health and well-being: a scoping review

**DOI:** 10.1186/s12913-018-3219-2

**Published:** 2018-06-28

**Authors:** Ingrid Larsson, Carin Staland-Nyman, Petra Svedberg, Jens M. Nygren, Ing-Marie Carlsson

**Affiliations:** School of Health and Welfare, Halmstad University, Box 823, S-30118 Halmstad, Sweden

**Keywords:** Children, Intervention, Participatory approach, Scoping review, User involvement, Young people

## Abstract

**Background:**

Greater interest is being shown in participatory approaches, especially in research on interventions that concern children and young people’s health and well-being. Although participatory approaches have user involvement in common, they differ in terms of the explicit guidance on how to actually involve and engage children and young people in health research. The aim of this scoping review was to systematically map recent research involving children and young people in the development of interventions targeting issues of health and well-being.

**Methods:**

An interpretative scoping literature review based on: a scientific literature search in (health and social science) databases, reference lists, a manual search in key journals and contact with existing networks was conducted. A total of 4458 references were identified through the literature search, of which 41 studies published between 2000 and 2017 were included in the review. The target population was children and young people under 25 years old. Level of participation was categorized according to Shier’s Pathways to Participation Model.

**Results:**

The review showed that participatory approaches were most often used in the development of interventions in school settings and in community and healthcare settings and on issues concerning support in lifestyle or in managing illness or disease. The level of participation varied from children and young people taking part just as active informants, through stages of greater participation both in quantitative and qualitative terms, to children and young people becoming an active agent involved as a co-researcher where the research process was shaped by views of a higher level of mutuality. Most of the studies were categorised at a medium level and only three studies were judged to involve the children and young people at the highest level.

**Conclusions:**

This scoping review showed that work remains in enabling children and young people to influence the development of interventions targeting health and well-being. In relation to level of sustainability in the interventions, it is relevant that goals, strategies and processes are formulated by those who can gain from the interventions. Participatory approaches aiming for a higher level of participation where children and young people work together with the researchers in partnerships are thus warranted.

## Background

A growing body of literature and health research policies has, in recent decades, emphasized the importance of more participatory approaches in health research that include views, knowledge, experiences and actions from those who are in focus of the research. Similarly, it has been highlighted that children and young people should be involved in co-creating new knowledge [1–6]. Although participatory approaches have user involvement in common, they differ in terms of the explicit guidance on how to actually involve and engage children and young people in health research [7, 8]. Many factors can challenge children’s and young people’s involvement, such as an underestimation of children’s competence to participate in research [1], attitudes that child involvement might adversely affect the quality of the research [9–11] or fear that participation might harm the child [2, 9–11]. As a result, children are usually merely involved as subjects in research and not as active research partners [3, 5]. Most health research for children and young people is thus primarily based on the involvement of parents, caregivers, and other stakeholders. However, such stakeholder perspectives cannot replace the qualities that come with genuine participation by the children and young people themselves [3, 5, 12, 13].

Children as social actors were emphasized by the Convention on the Rights of Children in 1989 [14]. The principles of the convention are relevant for how researchers relate to children’s participation and serve as a standard for how integration and assessment of children’s participation in research should be planned and assessed. Increased emphasis in health research on the need to develop solutions to improve the health of children and young people’s thus also requires that children are viewed as important partners in co-creating such solutions [15], providing both relevant knowledge for the design of solutions and guidance and planning for evaluation and implementation in practice [16]. When co-production approaches are used in health research, they increase the likelihood of developing more effective and efficient interventions that more precisely targets health and well-being issues among children and young people. This, however, requires a shift in the way research is designed, communicated and performed [17] and necessitates that children and young people are recognized as experts with the capability of contributing with unique experiences and knowledge [8]. However, it is difficult to classify and evaluate health research in ways that shows both the level of children and young people’s participation during the research process and its relation to the impact of the research and translation into practice [18]. In the application of participatory research it is important to distinguish three overall stages of participation [6], that differ both in principle and in the way they can be translated into practical implementation in health research. These stages are; nonparticipation, consultative participation, and collaborative participation. In the stage of nonparticipation, children are either not involved at all or are involved in ways that have no real impact on the research or that give a false semblance of partnership and sharing of power. At the stage of consultative participation, adults acknowledge the expertise of children and involve them in sharing their views and experiences, primarily through interviews or questionnaires. However, such approaches are neither giving the children control over the focus of the research or influence over the analysis or interpretation of data. At the collaborative stage, children are not only involved as experts but also take part at various degrees in initiation, planning, analysis and dissemination of the research. The partnership between the researcher and the children at this level is based on both trust and shared decisions [6]. Models that describe how participation can occur at different levels of intensity and quality are valuable as benchmarks in the planning of research and as guidance for evaluation of participation. Hart’s Ladder (1992) [19] adapted from Arnstein’s work [20] and Shier’s Pathways to Participation [21] are commonly used models when developing and evaluating children and young people’s participation in projects. Both models can be suitable when evaluating children and young people’s level of participation in research. In this scoping literature review, however, we decided to use Shier’s model [21] based on this model providing a more practical framework for planning and evaluating children and young people’s participation in practice. Shier’s model combines five levels of participation and three stages of commitment at each level describing the child’s transition from a passive actor towards having a partnership position where the child and the adult have equal positions [21].

Despite the increasing expectations that children and young people are involved as partners in health research, and despite that models for discriminating between different levels of participation and how to tackle challenges to participation are available, there is still considerable uncertainty among researchers about how to optimally provide opportunities for involving children and young people in health research. The aim of this scoping review was thus to systematically map recent research involving children and young people in the development of interventions targeting issues of health and well-being. The specific objectives were to; a) identify the extent of recent research using participatory approaches to involve children and young people in the development of interventions targeting health and wellbeing, b) grade the level of participation in such participatory approaches, and c) identify areas for further research.

## Methods

### Study design

An interpretative scoping literature review, based on the framework of Arksey and O’Malley [22], was chosen for the study design. A scoping review contributes a systematic knowledge synthesis in a defined area based on an explorative research question with the aim of mapping key concepts, available evidence, and gaps in the research [23]. Scoping reviews are useful for summarizing and describing data from a wider range of fields and disciplines and for identifying gaps in the literature, and thus the quality of the studies are not the focus of the evaluation. To enable replication and strengthen methodological rigour, this study follows the five-stage methodological framework of Arksey and O’Malley [22]; identifying the research question, identifying relevant studies, study selection, charting the data, and collating, summarising and reporting the results.

### Identifying the research question

The core question in this scoping review was: In which areas and to what extent were children and young people involved in the development of interventions targeting children and young people in health and well-being. The definition of children, in this scoping review, was a person under 18 years old, which is in line with The United Nations Conventions of the Rights of the Child [14], while young people are referred to a person between 15 and 24 years old, in accordance with The United Nations [24].

### Identifying relevant studies

The evidence was searched by way of electronic databases, reference lists, hand-searches of key journals and contact with existing networks. With the help of experienced librarians an electronic database search incorporating Academic Search Elite, CINAHL, ERIC, Medline, PsycInfo, Sociological Abstracts and SportDiscus was conducted in December 2014, updated in April 2015 and finally updated in December 2017. Different techniques and terms were used for expanding and narrowing searches, including search tools such as medical subject headings (MESH), Boolean operators and Truncation. Single and combined search terms included the key words: “child”, “adolescent”, “participate”, “participation”, “collaboration”, “involve”, “involvement” and “intervention”. Relevant publications were defined as any empirical peer-reviewed paper. No limitations were set in terms of the publications date, as no previous review focusing on children and young people’s participation in the development of interventions had been performed. English was chosen as the language for the database search, as it is the most commonly used language in scientific journals. Inclusion criteria were: articles with children and young people under 25 years old; articles in which children and young people participate in one or more levels of the development of an intervention aimed at children and young people in health and well-being. Exclusion criteria were: articles in which children and young people were only participants in an intervention or evaluated an intervention.

### Study selection

**Fig. 1:**
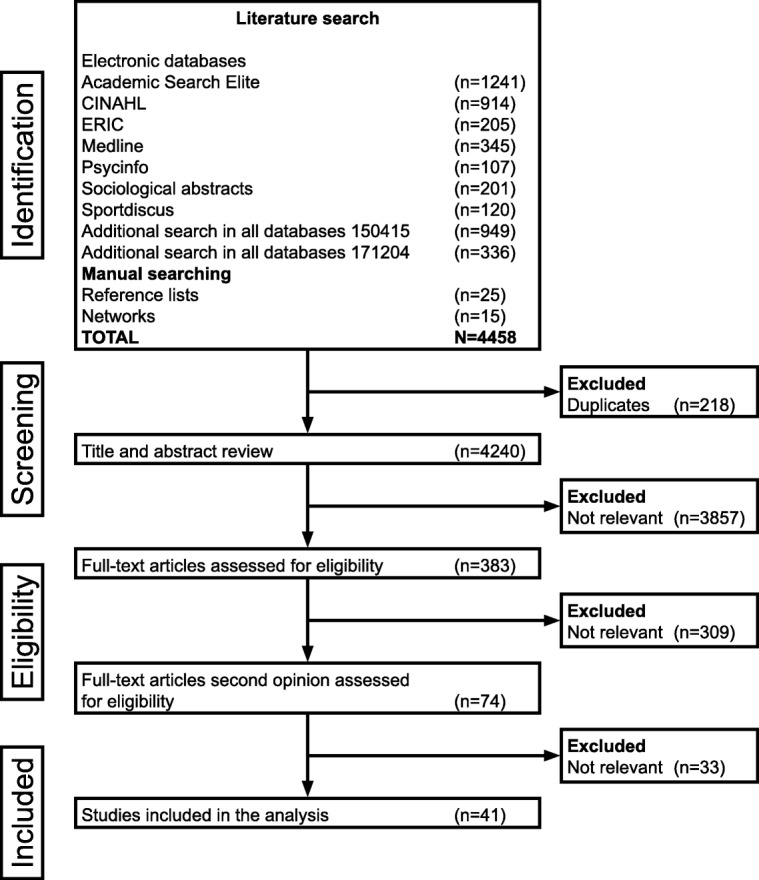
Flowchart of literature search and selection

The searches identified 4458 articles, which were catalogued in EndNote®. Duplicates (*n* = 218) were removed by automation, supplemented by manual checking. An initial scan of title and abstracts identified large numbers (*n* = 3857) of irrelevant studies. A total of 383 articles were retrieved and read in full text by the authors. Seventy four studies were identified as potentially relevant after an elimination process based on the inclusion and exclusion criteria. These articles were reviewed by three of the authors (IL, CSN, IMC) and by consensus a final decision was made on which articles to include. The development of a particular intervention was sometimes described in a number of articles and in these cases only one of these articles describing the intervention was included in this scoping review. In some articles, when the target group for the intervention was children and young people, the development of the interventions was poorly described, but it appeared that the researchers developed the intervention together with parents, health professionals or other adults. Articles were excluded if children and young people participated in the implementation of an intervention or in the evaluation of interventions with the specific aim of modifying an intervention designed by the researchers. A total of 41 articles were finally included in the scoping review (Fig. 1).

### Data charting and collation

The authors created a data charting form which included: reference (author, year), country, key aims, number and age of children and young people, type of intervention, aim of participation, and finally in which parts of the developmental process the included participants were involved. The authors (IL, CSN, IMC) first extracted data independently and then met to determine whether the data extractions were consistent with the aim of the study and the research questions (Table 1).

**Table 1:** Characteristics of the articles included in the scoping review, based on level of participation

Reference Country Setting	Aim	Participants					Intervention	Participating in	Level of Participation (Shier, 2001)
		N	Age (years)	Sex		Kind of participant			
				Female	Male				
Goold et al., 2006 [50] UK School	To develop an intervention for use in sexual health promotion.	16	13–14	12	4	Students	An Interactive Multimedia Learning Environment for sexual health interventions	Focus group interviews	Level 2 The participants were informants
Hawkins et al., 2016 [51] UK School	To identify the key components, feasibility and acceptability of a Group Motivational Interviewing (GMI) intervention for promoting health behaviours in schools with focus on alcohol consumption	12* * In additional 8 teatchers participated	12–14	N/S	N/S	Students in secondary school	A health promotion interventions focus on alcohol consumption Group Motivational Interviewing intervention (GMI)	Focus group interviews	Level 2 The participants were informants
Wind et al., 2005 [60] Belgium and Netherlands School	To identify personal beliefs and motivations as well as possible environmental factors that are related to schoolchildren’s fruit and vegetable intake	92	10–12	47	45	Schoolchildren in primary school	A Pro Children intervention	Focus group interviews	Level 2 The participants were informants
Akard et al., 2013 [25] USA Healthcare	To develop a legacy-making intervention for children with cancer	Part I: 8 Part II: 1	7–12 N/S	N/S N/S	N/S N/S	Patients with cancer	A legacy intervention/memory making for parents and their children with cancer	Part I: Individual interviews Part II: Individual interview	Level 3 The participants were informants
Arora et al., 2013 [61] India Community	To inform the development and test the appropriateness of project ACTIVITY’s intervention model	148* *In addition 46 adults participated	10–19	yes	yes	Young people living in slums and resettled colonies.	A project to Advance Cessation of Tobacco in Vulnerable Indian Tobacco Consuming Youth (ACTIVITY)	Focus group interviews	Level 3 The participants were informants
Beaulac et al., 2009 [26] Canada Community	To develop a better understanding of barriers and facilitators to adolescent participation in physical activity and to identify preferences and concerns regarding the characteristics of a new physical activity programme	17* *In addition 13 mothers participated	11–14	10	7	Young people from multicultural neighbourhoods	An intervention in physical activity programme with hip hop dance	Focus group interviews	Level 3 The participants were informants
Beaulieu et al., 2012 [27] Canada School	To identify the determinants of students staying for lunch at a high school to develop interventions promoting the targeted behaviour.	Part I & II: 153	12–17	yes	yes	High school students	An intervention programme to encourage high school students to stay in school for lunch.	Part I: Survey Part II: Focus group interviews	Level 3 The participants were informants
Biltoft-Jensen et la., 2014 [47] Denmark School	To describe the development and formative evaluation of web-based Dietary Assessment Software for Children (WebDASC).	Part I: 20 Part II: 5 Part III: 70	8–11 8 8–11	N/S N/S N/S	N/S N/S N/S	Children with different gender, ethnicity and background	An intervention programme with a self-administrated dietary assessment (WebDASC)	Part I: Focus group interviews Part II: A pilot test with the think-aloud method Part III: Usability tests of a prototype	Level 3 The participants were informants
Cafazzoet al., 2012 [28] Canada Healthcare	To design, develop, and pilot a mHealth intervention for the management of type 1 diabetes in adolescents	Part I: 6* Part II: 20 *In addition parents and clinical team participated	12–16	N/S 10	N/S 10	Patients with type 1 diabetes	A home and community- based diabetes tele management system (mHealth diabetes app)	Part I: Individual interviews Part II: Clinical pilot test of the intervention	Level 3 The participants were informants
Caldwell et al., 2004 [29] USA Community	To develop a conceptual model to inform intervention development and implementation to strengthen nonresident African American father and son relationships	Part I, II N/S* *In total 77 African American sons, fathers and mothers participated	8–12	0	yes	African American boys	A family-centred, culturally relevant and gender specific intervention to strengthen relationships between fathers and sons in order to reduce substance use and violent behaviour and to prevent early sexual invitations	Part I: Focus group interviews Part II: Focus group interviews to refine the intervention	Level 3 The participants were informants
Corder et al., 2015 [48] UK School	To develop a physical activity promotion intervention for adolescents	Part I 480 Part II 31	13–14 16–18	yes 11	yes 20	Students	An intervention to increase physical activity among adolescents (GoActive intervention)	Part I Survey Part II Focus groups interviews and individual interviews	Level 3 The participants were informants
Elf et al., 2012 [49] Sweden Healthcare	To reveal young carers’ views of design of a web-based support system (WBSS)	Part I: 12 Part II: 8	16–25 17–25	8 4	4 4	Young carers supporting someone with mental illness	A web-based support system (a web-site)	Part I: Individual interviews or a focus group Part II: Video recording, design meetings	Level 3 The participants were informants
Goodkind et al., 2012 [32] Mexico Community	To create and pilot test a prevention/healing intervention model for American Indian youth and their families	18	7–17	14	4	American Indian youths	Our Life Intervention: An intervention to promote mental health of American Indian Youth	Focus group interviews	Level 3 The participants were informants
Grant et al., 2014 [33] USA School	To develop a new intervention for low-income urban youth at risk of negative academic outcomes	Part I, II, III: N/S* *In addition parents, school staff, community leaders participated	N/S	N/S	N/S	Students in 8th grade	Cities Mentor Project: An intervention to improve academic outcomes for low-income urban youth	Part I: Focus group interviews Part II: Community advisory board meetings Part III: Observations of feasibility of the intervention	Level 3 The participants were informants
Greene et al., 2016 [34] USA School	To readjust an alcohol-targeted high school media literacy intervention by feasibility test of two versions of the YMD	Part I: 148 Part II: 20 Part III: 13	14–16	104 8 6	44 12 7	Students	An intervention targets high school student alcohol use by the Youth Message development (YMD) curriculum	Part I: Pilot testing of two versions Part II: Individual interviews Part III: Focus group interviews	Level 3 The participants were informants
Kong et al., 2012 [36] Mexico School	To design a school-based obesity prevention programme	7* * In additional 8 parents participated	N/S	N/S	N/S	High school students with overweight	A school-based intervention for high schools to promote healthy eating and physical activity	Individual interviews	Level 3 The participants were informants
Lowes et al., 2011 [53] UK Healthcare	To describe the active involvement of stakeholders in the development of a research intervention	Part I, II: N/S* * In total 28 teenagers, parents, adult patients, and professionals participated	N/S	N/S	N/S	Teenagers with diabetes type 1	A Psycho-social Intervention in Children and Teenagers Experiencing Diabetes (DEPICTED)	Part I: Focus group interviews Part II: Experimental consultations to evaluate and refine the intervention	Level 3 The participants were informants
Lutenbacher et al., 2002 [37] USA Community	To identify practical components of decision-making for a youth violence prevention programme planning and to identify differences in decision-making across various provider sectors of the community	10* *In additional 73 adult people participated	14–18	7	3	Youths	A Youth violence preventive programme	Focus group interviews	Level 3 The participants were informants
Maynard et al., 2009 [54] UK School	To assess the feasibility, efficacy and cultural acceptability of child- and family-based interventions to reduce risk factors for childhood and adolescent obesity among ethnic minorities	Part I: 70* Part II, III: N/S* * In addition 43 parents, 12 other adults participated	8–13	N/S N/S	N/S N/S	Pupils with different ethnicity	The DEAL, Diet and Activity Living	Part I: Focus group interviews Part II: Photographs of the intervention piloting Part III: Focus group interviews and written evaluations	Level 3 The participants were informants
Milnes et al., 2013 [55] UK Healthcare	To develop an evidence-based pre-consultation guide for young people to use prior to an asthma review with a practice nurse.	Part I: 6 Part II: 8	16–18 13–18	6 5	0 3	Young people with asthma	A pre consulting guide to promote communication in consultations	Part I: Expert panel discussions via e-mail and social networking site Part II: Focus groups interviews	Level 3 The participants were informants
Mishra et al., 2005 [62] India School	To plan interventions to prevent and control tobacco use among youth in India as part of Project MYTRI	435	10–16	181	254	Students in government and private schools	The MYTRI project (Mobilising youth for the tobacco related initiatives in India)	Focus group interviews	Level 3 The participants were informants
Morales-Campos et al., 2015 [38] USA Community	To develop an community-based intervention for physical activity among Hispanic girls	Part I-IV: 40	11–14	40	0	Hispanic middle school girls	Physical Activity Partnership for Girls (PG) project	Participatory Photo Mapping (PPM) Part I: Photo walking tours in groups Part II: Photo sharing group discussion Part III: Creating a photo poster Part IV: Presenting the photo poster	Level 3 The participants were informants
Power et al., 2004 [64] Zimbabwe School	To determine the feasibility and acceptability of conducting a community randomised trial of an adolescent reproductive health intervention in rural Zimbabwe.	N/S* * In addition parents, school staff, community leaders and healthcare providers participated	12–17	N/S	N/S	Students in secondary schools	A Adolescent reproductive health intervention	Focus group interviews Individual interviews Observations	Level 3 The participants were informants
Power et al., 2010 [39] USA School	To inform the development of a multi-strategy, school-based obesity prevention programme for early adolescences	16* * In addition 6 parents and 11 teachers participated	12–14	11	5	Students	A school-based prevention programme for adolescent obesity The Teen Eating and Activity Mentoring in Schools project (TEAMS)	Focus group interviews	Level 3 The participants were informants
Raghupathy et al. 2012 [40] USA School	To describe the process by which an existing evidence-based culturally relevant drug prevention intervention was transformed into a low cost computerised intervention (HAWK a computer and web-based intervention	Part I: N/S* Part II: about 45* * In addition community experts, scientists, school staff participated	N/S 11–13	N/S N/S	N/S N/S	Native American youth in reservations and rural locations	A computer as well as a web-based drug abuse prevention intervention, HAWK^2^ (Honoring Ancient Wisdom and Knowledge)	Part I: Video recording, photographs and script-making Part II: Reviewing the prototype	Level 3 The participants were informants
Reinaerts et al., 2006 [56] Netherlands School	To explore factors that are associated with children’s fruit and vegetable (F&V) intake, to develop a school-based intervention to increase their F&V consumption	104* *In addition 38 parents participated	3–14	50	54	Pupils in primary schools	A school-based intervention to increase fruit and vegetable consumption among primary school children	Focus group interviews	Level 3 The participants were informants
Resnicow et al., 2000 [41] USA Community	To develop and implement a nutrition and physical activity intervention	17	N/S	17	0	Overweight African American adolescent	A nutrition intervention for overweight African American adolescent women (Go Girls)	Focus group interviews	Level 3 The participants were informants
Sockolow et al. (2017) [43] USA Community	To develop and employ an innovative approach, Experiential Participatory and Interactive Knowledge Elecitation (EPIKE) to generate design and content requirements for a psychoeducational mobile health (mHealth) intervention	Part I, II: 22 Part III: 9	13–17	15 6	7 3	African Americans and Latinos adolescents	A mobile health (MHEalth) psychoeducational intervention for at-risk adolescent	Part I: Role play Part II: Design sessions Part III: Testing the prototype	Level 3 The participants were informants
Sorensen et al., 2004 [44] USA Community	To describe the formative research process that was used to develop a tobacco control intervention for working teens	Part I: 41 Part II: 375	N/S 15–18	N/S 179	N/S 196	Teens both smokers and non-smokers	A worksite-based tobacco control intervention for working teens SMART, Teens Against the Risks of Tobacco	Part I: Focus group interviews Part II: Cross-sectional survey	Level 3 The participants were informants
Wright et al., (2016) [65] Australia Community	To develop and investigate the feasibility and acceptability of mobile phone-delivered data collection and intervention for young people during drinking events.	Part I: 42 Part II: 40 Part III: 40	18–25	21 yes yes	21 yes yes	Youths	An Mobile-Phone delivered Ecological Momentary Assessment (EMA) intervention for young people during drinking events	Part I: Workshops Part II: Testing the intervention Part II: Survey and in-depth interviews	Level 3 The participants were informants
Young et al., 2006 [45] USA School	To describe how formative research was used to design the Trial of Activity for Adolescent Girls (TAAG) intervention	Part I: 130 Part II: 87 Part III: 77 Phase IV: 100	N/S	130 87 0 100	0 0 77 0	Schoolchildren in 6–8 grade	An intervention to reduce decline of physical activity: Trial of Activity for Adolescent Girls (TAAG) intervention	Part I: Survey Part II: Focus group interviews Part III: Focus group interviews Part IV: Focus group interviews	Level 3 The participants were informants
Arvidsson et al. 2016 [46] Sweden Healthcare	To redesign Sisom for use on mobile devices and to validate and adapt it for use in a Swedish population of children with cancer	10	6–11	4	6	Healthy children and children with cancer	An interactive computer-based assessment and communication tool to give children with cancer a “voice” in their care (SISOM 2)	Part I: Observations and Think aloud Part II: Individual interviews Part III: Drawing and writing	Level 4 The participants were informants, designers and evaluators of the intervention.
Cottrell et al., 2010 [30] USA Community	To describes the process used to identify community health beliefs and the development of theoretically based materials to increase participation	Part I: 92 Part II: 240 Part III: 5	mean 11 mean 10 mean 11	N/S N/S N/S	N/S N/S N/S	Schoolchildren in fourth and fifth grade	The Coronary Artery Risk Detection in Appalachian Communities (CARDIAC) to reduce children’s future cardio-vascular risk by implementing a school-based screening programme	Part I: Individual and focus group interviews to expand and revise a Health Belief Questionnaire (HBQ) Part II: Survey and the findings led to design and development of intervention Part III: Focus group interviews. The intervention material revised according to the feedback	Level 4 The participants were informants, innovators and evaluators of the intervention.
Jenkins et al. 2016 [35] Canada Community	To develop the CollaboraKTion Framwork intervention for Community-Based Knowledge Translation to strengthening population health	10	13–18	N/S	N/S	Young people	An evidence-informed mental health promotion intervention as an web app	Part I: Weekly videoconference meetings Part II: In-person meetings Part III: Training sessions	Level 4 The participants were informants, designers and innovators of the intervention.
Ruland et al., 2008 [57] Norway Healthcare	To describe design challenges in the development of a clinical support tool for seriously ill children	Part I: 12* Part II: 5* Part III: 14* Part IV: 10* *A total number of 41 children (29 healthy children and 12 children with cancer)	9–11 9–11 8–12 7–11	7 2 4+4 N/S+4	5 3 4+2 N/S+2	Healthy children Healthy children Healthy children+Children with cancer Healthy children+Children with cancer	A clinical support tool for seriously ill children with cancer to improve patient communication (SISOM)	Part I: Design groups with a think-aloud method, role plays Part II: Individual interviews and observations Part III: Individual interviews with selection of meaningful child-friendly terms used in the system and tested the prototype Part IV: Usability tests with observations and video recording as end-users and the prototype was developed further	Level 4 The participants were informants, designers, innovators evaluators and testers of the intervention
Schultz et al., 2001 [42] USA Healthcare	To develop and evaluate theory-based interventions designed to change sexual behaviour and promote safer sex practices of HIV seropositive young men and adolescents with haemophilia	Part I: 59 Part II: 97* *In addition parents to respondents under 18 were also surveyed	13–23	N/S N/S	N/S N/S	Adolescents.	A Haemophilia Behavioural Intervention Evaluation Projects (HBIEP)	Part I: Individual interviews to develop a questionnaire Part II: Survey with the developed questionnaire and the findings of this determined the focus for the interventions	Level 4 The participants were informants and evaluators of the intervention
Stålberg et al., 2016 [58] Sverige Healthcare	To develop an interactive application to facilitate young children’s participation in healthcare situations	Part I: 43 Part II: 9 Part II: 33 Part IV: 9	3–5	22 7 13 5	21 2 20 4	Children from three settings; preschool, primary health care clinic and outpatient clinic	An application to facilitate young children’s participation in healthcare situations, the Inter-Active Communication Tool for Activities (IACTA)	Part I: Individual interviews and drawings Part II: Tested and evaluated a paper prototype Part III: Tested and evaluated the first interactive prototype Part IV: Tested and evaluated the second interactive prototype	Level 4 The participants were informants, designers and testers of the intervention
Tan et al., 2011 [63] Singapore School	To describe the Creativity, Activities, Learnability, Storylines, Interactivity, Usability and Multimodality (CALSIUM) framework to elicit children’s contributions and perspectives in the design of an online game for enhancing social skills of children.	Part I, II, III: 12	10	6	6	Students in primary school	A computer game to increase social skills (Socialdrome)	Part I: Testing a prototype Part II: Focus group interviews Part III: Workshops to design a prototype using storyboarding	Level 4 The participants were informants, designers and testers of the intervention
Garofalo et al., 2012 [31] USA Community	To develop and pilot test a homegrown intervention addressing HIV prevention needs of young transgender women	Part I: N/S Part II: 8 Part III: 7	N/S 16–24 16–24	yes 8 7	0 0 0	Young transgender women	An intervention to prevent HIV, the Life Skills	Part 1: Initiated the intervention with assistance of a research team Part II: Focus group interviews refined the intervention Part III: Pilot group tested the revised intervention and gave feedback on the content and logistical aspects of intervention delivery	Level 5 The participants were informants, designers, innovators evaluators and testers of the intervention
Kime et al., 2013 [52] UK Healthcare	To involve young people in developing a self-care intervention for young people with type 1 diabetes or asthma.	Part I: 87* Part II: N/S* * In addition 7 young adult facilitators (3 with diabetes type 1 and 4 with asthma), aged 18–25 years participated	12–17	40 N/S	47 N/S	Patients with diabetes type 1 (*n* = 41) and with asthma (*n* = 46)	A self-care intervention for young people to better manage diabetes type 1 or asthma and improve their overall quality of life	Part I: Focus group interviews with facilitators, aged 18–25 with the same diagnosis. Part II: Design workshops with participants working alongside the researchers to decide the format of the intervention	Level 5 The participants were informants, designers, innovators evaluators and testers of the intervention
Wärnestål et al., 2017 [59] Sweden Healthcare	To develop digital peer support services (DPS) directed towards children surviving cancer in order to facilitate health-promoting social connectedness to other children with similar experiences.	Part I: 15* Part II: 5 Part III: N/S* * In addition there were 9 Stakeholders/ healthcare professionals and 4 parents in part II, III	8–12 11–13 N/S	15 2 N/S	0 3 N/S	Children with cancer	A Digital peer support services (DPS)	Part I: Focus group interviews Part II: Design workshops with pairs of one participant and one researcher or one participant and one professional designers Part III: Focus groups interview	Level 5 The participants were informants, designers, innovators evaluators and testers of the intervention

N/S = not specified in the article

Yes = Included in the article but the number of participants is not specified

### Collating, Summarising and reporting findings

In order to grade the level of participation in the analysis, we summarised and categorised the data in accordance with Shier’s Pathways to Participation Model [21]. This model includes the following five levels of participation:

- Level 1, *Children and young people are listened to*, requires that researchers listen to the children and young people when the children and young people take the initiative to express their views. In this stage the children and young people can thus be seen as responsible for taking active initiatives in order to participate. At level 2, where *children and young people are supported when expressing their views*, the initiative and responsibility is moved to the researcher who has to find and facilitate ways for the children and young people to express their views and by this enable the children and young people’s participation. At level 3, the degree of participation is extended and *children and young people’s views* (explicitly or non-explicitly expressed) *are taken into account*. This level is distinguished from level two in that children and young people’s views are not only listened to or asked for but are also seriously taken into account with the aim of having influence. At level 4, *children and young people are involved in decision-making processes.* This level and level 5, where children and *young people share power and responsibility in decision-making* are characterised by a successive transition from children and young people seen as consultants to a stage where they obtain a position of power. These are characterised by the willingness of researcher to share or give up their power in favour of children and young people’s contribution. In moving up the levels (1–5) the model describes the children or young people moving from being a passive informant to an active agent towards a partnership position where researchers and children or young people are in an equal position.

In order to categorise the level of children and young people’s participation, the included studies were judged overall based on a) quantitative aspects of participation i.e. number of activities or stages in the developmental process that included children and young people and b) qualitative aspects i.e. to what extent such involvement was based on reciprocity concerning influence, power and decision-making in the developmental process. In order to strengthen the validity in this process the authors (IL, CSN, IMC), who were multidisciplinary, independently analysed the children and young people’s level of participation in each intervention in accordance with Shier’s five-stage framework [21]. The authors discussed and compared their analysis and level of participation in the articles until consensus was reached.

## Results

Our overall aim with this scoping review was to grade children and young people’s participation in the development of interventions. To enable this goal and to gain an overall view of the field we needed to make sense of our complex findings. Therefore, in addition to displaying the number of articles, we also found it useful to map the research fields according to general characteristics of the included articles such as the settings and countries were the research took place. It was also considered of interest to focus on the interventions made and the methodological characteristics of the included articles, i.e. the actions agreed as the result of the research and the types of practices that were needed to promote children and young people to participate in the research field. Furthermore, we used Shier’s [21] model to analyse and grade our findings according to the children and young people’s level of committed participation in the research. To allow this view of the field to be seen, the findings are illustrated in three areas; general characteristics of the included articles, methodological characteristics of the included articles and children and young people’s level of participation in the development of interventions in the included articles, with a combination of texts, tables, and figures.

### General characteristics of the included articles

There was a total of 41 articles included in this scoping review. These were published between 2000 and 2017 and were from different parts of the world. A majority of the studies, 21, were conducted in North and Central America [25–45] and 15 in Europe [46–60]. A small minority of the studies were thus undertaken in the other continents, Asia [61–63], Africa [64] and Australia [65]. The participants in the included articles varied in age between 3 and 25. Four of the included studies did not, however, explicitly declare the participants’ age and instead used terms such as teenager or teens [44, 53], adolescents [41] and students [36]. Another way of expressing the participants’ “age” was to refer to which grade or school system the participants were in when the research took place [33, 45].

Furthermore, the included articles in this scoping review varied according to research settings. We categorised three broad settings, 13 were conducted in community settings [26, 29–32, 35, 37, 38, 41, 43, 44, 61, 65], a further 11 in healthcare settings [25, 28, 42, 46, 49, 52, 53, 55, 57–59], and 17 in school settings [27, 33, 34, 36, 39, 40, 45, 47, 48, 50, 51, 54, 56, 60, 62–64].

**Fig. 2:**
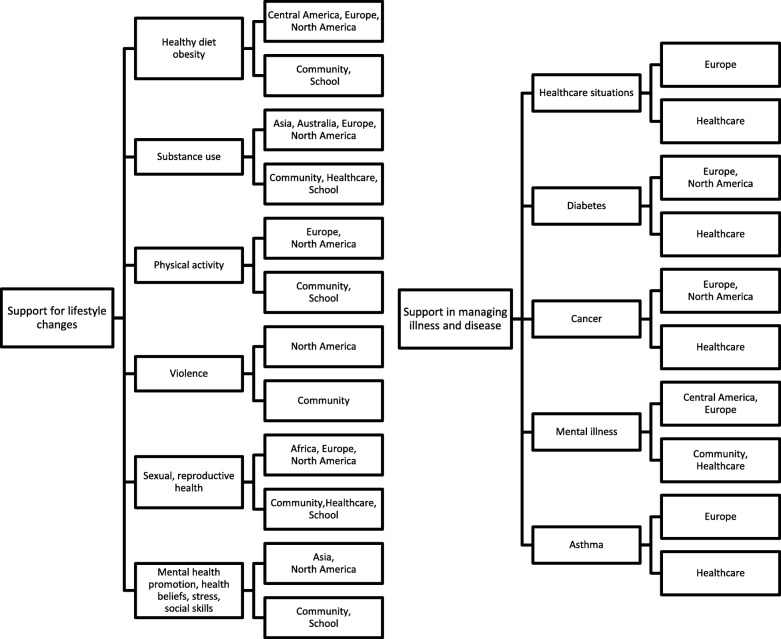
Focus and issues of the interventions and the continent and research settings

The reviewed articles had two different foci for the developed interventions, support for lifestyle changes and support in managing illness and disease (Fig. 2). However, both of these foci were supporting interventions concerning health and well-being. The main areas in 30 articles were supportive lifestyle interventions and concerned: a healthy diet and obesity [27, 36, 39, 41, 47, 54, 56, 60], physical activity [26, 36, 38, 39, 41, 45, 48, 54], substance abuse such as, alcohol, tobacco and drug use [29, 34, 40, 43, 44, 51, 61, 62, 65], sexual and reproductive health [29, 31, 42, 43, 50, 64], violence [29, 37], stress [33], social skills [63], health beliefs [30] and mental health promotion [35]. In addition to lifestyle issues, eleven of the articles considered issues that supported children and young people with their illness or disease. These were supportive interventions to manage healthcare situations [58], support children and young people with cancer [25, 46, 57, 59], diabetes [28, 52, 53], mental illness [32, 49], and asthma [52, 55].

### Methodological characteristics of the included articles

Most of the studies included in the scoping review have used interviews as a data collection method to give a voice to the participants. Focus group interviews was the most common data collection method [26, 27, 29–34, 37, 39, 41, 44, 45, 47–56, 59–64] while other studies have used individual interviews [25, 28, 30, 34, 36, 42, 46, 48, 49, 57, 58, 64] with children and young people. However, seven of the studies reported including both interviews and surveys to involve the children and young people’s views in the development of the intervention [27, 30, 42, 44, 45, 48, 65]. As a supplement to these more traditional data collection methods there were also research designs that included a range of innovative methods, such as video recordings [40, 49, 57] photographs [38, 40, 54] drawings and texts [46, 58, 59] advisory boards [33], e-mail and a social networking site [55], observations [57, 64], script-making [40, 43], storyboarding [63], active and spontaneous role play [43], and videoconferences and in-person meetings [35].

The interventions were sometimes tested for feasibility and usability and a wide range of methods were used, including face validity [25] think-aloud methods [43, 46, 47, 57], interviews [31, 32, 34, 65] and observations [33, 57]. Written feedback [27, 31, 34, 54], workshops [52, 59, 65] and a mobile survey [65] were also used.

### Children and young people’s level of participation in the development of interventions

**Fig. 3:**
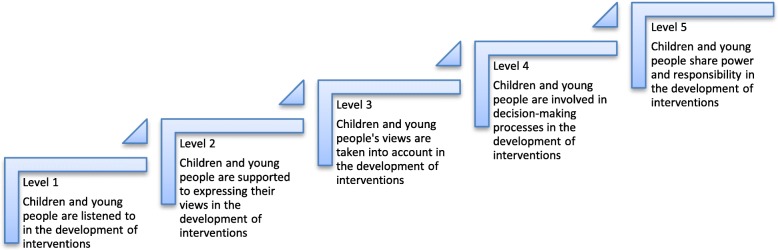
Applied description of Shier’s Pathways to Participation Model in relation to included studies in the current scoping review

The articles were also graded according to the children and young people’s level of participation in the development of the interventions. We used Shier’s model, which contains five levels of participation [21], as a framework to map our findings (Fig. 3).

All studies regardless of design were graded from level one to level five. However, due to the inclusion criteria in this scoping review, where children and young people had to be involved in the development of an intervention, all the included articles met the criteria for the second level according to Shier’s model [21]. Level one is thus not further described in the findings section. There was, however, a variation in how clearly and detailed the authors described the research process as to how the voice of the children and young people influenced the development of the interventions.

The findings showed a variation in the articles in terms of the children and young people’s level of participation. Furthermore, this participation varied both in quantitative and qualitative terms from just being an active informant to an active agent taking part in more steps in the research process as a co- researcher. When the research process was shaped by views of a higher level of mutuality the participants were enabled to share power and responsibility in the research process.

#### Level 2. Children and young people are supported to express their views in the development of interventions

This was the lowest level for participation found in this scoping review. The children and young people simply had a participatory role as informants in the research process at this level. Only three articles [50, 51, 60] in this scoping review were analysed as only meeting the criteria for level two. The low rating for these articles was due to the researchers only describing that they had supported the children and young people to express their views and that they had facilitated ways for listening to the participants. The researchers did not, however, describe how the information was used. Furthermore, these articles only stated that the performed focus groups were to inform the development of an intervention but the researchers did not explicitly describe the ways in which the information affects the intervention [50, 51, 60]. It is unclear if the participants’ voices were taken into account or not thus leaving the reader with unanswered questions.

#### Level 3. Children and young people’s views are taken into account in the development of interventions

The vast majority, in total 28, of the included scoping-review articles, met the criteria for level 3 [25–29, 32–34, 36–41, 43–45, 47–49, 53–56, 61, 62, 64, 65]. The distinction between this third level and the previous one was that the children and young people’s views were not only asked for and listened to, but their voice was also seriously taken into account with the aim of influencing the further process of the development of the intervention, and that this was explicitly expressed in the article.

Expressions such as; “helped to develop the intervention”, “guided” or “informed” were used to describe the ways in which the children and young people’s views were taken into account at this level. We can therefore conclude that the children and young people were listened to and that they also had an impact on the development of the intervention. However, the words that explain the participatory part of the development are relatively vague leaving the reader uncertain as to how much of the information was used by the researchers.

#### Level 4. Children and young people are involved in the decision-making processes in the development of interventions

Seven of the 41 articles in this scoping review were considered to fulfil the criteria for the fourth level of participation [30, 35, 42, 46, 57, 58, 63]. To be able to attain this assessment the article had to explicitly describe that the researchers involved the participants in the decision-making process when the intervention was developed. This implies that the children and young people’s voice has an even greater level of importance and is taken into consideration. It is not sufficient for this level of participation that the researchers themselves choose which part of the information is to be used in the development. The children and young people’s voices need to instead be taken more seriously and there needs to be a successive transition, from just seeing the children and young people as consultants to a position where they have a much more important and extended position in the development of the intervention. In the articles at this level, the researchers explicitly expressed that the participants; “developed the design”, “determined the development” or “accounted and contributed to the design” as well as being “central collaborators*”* and “co-designers*”*.

#### Level 5. Children and young people share power and responsibility in the development of interventions

Only three of the included articles managed to reach the highest level in Shier’s model of participation [31, 52, 59]. Level 4, children and young people are involved in decision-making processes, and level 5, where children and young people share power and responsibility in decision-making are characterised by a successive transition from children and young people as consultants to a stage where they obtain a position of power. These are also characterised by the willingness of researchers to share or give up their power in favour of the children and young people’s contribution. In moving up the levels (1–5) the model describes the child or young person moving from a passive informant to an active agent towards a partnership position where researchers and children hold an equal position.

The three studies that achieved this level were more characterised by a willingness from the researchers to share the power to influence the developing process with the participants. The research idea in the study by Garafolo et al. [31] originated from the participants themselves. Young transgender women created the intervention with assistance of the research team. The feasibility of the intervention in this study was then tested and evaluated with the participants and their further suggestions were used to refine the intervention. The researchers and the participants in the study by Kime et al. [52], worked “side by side” at different stages of the research process. Finally, in the study by Wärnestål et al. [59], the researchers explicitly named the participants as “designers” and “innovators” and described the co-creation in developing the intervention during the whole process together with the children. This demonstrated that the children and young people had an extended role where they could influence the development. The designation as a designer also proved that the researchers were willing to share a certain amount of power over the developmental process. In addition, the participants were included in several of the research steps as informers, testers, evaluators and finally as informants about the usability of the intervention.

## Discussion

The need for and operationalization of more explicit participatory approaches in research where participants actively take part throughout the research process has increasingly been discussed. This review provides an overview of research during 2000–2017 with a focus on children and young people’s level of participation in research aiming at developing health and well-being interventions. Results from research from both the health and social sciences were included, which to our knowledge has not previously been performed. The main findings showed that the explicit level of participation in the included studies varied greatly, both in quantitative and qualitative terms, and that the designs and the methodologies used in order to increase the level of participation were generally poorly described. Even though studies reported an ambition to increase children and young people’s participation in the research process, actually doing so was more seldom supported by the results. The participants only took part as informants in a majority of the studies [25–29, 32–34, 36–41, 43–45, 47–51, 53–56, 60–62, 64, 65] and surprisingly few reached the higher levels of participation [30, 31, 35, 42, 46, 52, 57–59, 63] in Shier’s model [21]. These findings were somewhat unexpected since approaches, in which representatives from the target group formulate the needs and goals of an intervention, increase the level of sustainability of the outcomes [5, 66].

In line with the work of Reich et al. [67], the studies that reached the fourth [30, 35, 42, 46, 57, 58, 63] and the fifth [31, 52, 59] levels appeared to be less expert-driven. Furthermore, a specific feature of the studies on the fifth level was that the children and young people were co-researchers in all parts of the development process. For example, participants were described as designers or that they were working alongside the researchers. In one of the studies [59] working pairs of participants and researchers or professional designers were formed. These types of descriptions indicate a higher level of partnership mutuality and that a transfer of power, from researchers to participants, was facilitated and had possibly taken place. Broström maintains that research needs to include the participants at all stages of the process if the participants are to be recognised as co-researchers [68]. Consequently, participants also need to have an influence on the research questions and development of the interventions from the start to ensure that the intervention responds to their needs and takes their specific contextual situation into account [69]. It was only in the study by Garafolo, in this review, where it was reported that the participants had an opportunity to influence the intervention from the conceptual stage [31].

In spite of having participatory approaches as a common denominator, it was notable that the descriptions of the methods varied considerably as to how these approaches were carried out throughout the research process. Several studies had inadequate descriptions of the methods used and were therefore excluded at an early stage. Moreover, information concerning design and methodology were lacking or incomplete in some of the studies that met the criteria for inclusion. This is an important result since a more accurate and thorough methodological description could help revealing which factors, conceptions and assessments influenced the participatory approach in the reviewed studies. Moreover, the information could have been significant for the understanding of how different mechanisms in the research process were influenced by contextual factors as well as how they in turn informed the results of the developed intervention [70].

Levels of possible and expected participation will by nature vary with the age of the children as well as with their knowledge and experience of a certain issue or situation. Since previous studies have pointed out that children’s capabilities are often underestimated [1, 4] it was particularly interesting, that one of the studies reaching level four, involved children as young as three to 5 years of age [58]. Appropriate and mixed data collection strategies are necessary for successful inclusion of children and young people as active research participants [71] and indeed those studies that reached the highest level of participation used a variety of data collection strategies. In addition to interviews or surveys, the most common methods of data collection for studies at higher levels of participation, were methods where children could be active and express themselves in other ways than verbal ones [30, 31, 35, 42, 46, 52, 57–59, 63]. Such methods could for example include drawing, painting, writing [72], theatre [73], photography, filming [74] workshops, storytelling using software and digital tools, and mapmaking [73]. Using such methods at different stages in the research process does not in itself guarantee a participatory approach [74, 75], but they can promote the participants’ sense of control and thereby enable them to take a more active part in the research process [73]. Methodology in itself can thus push a traditional top-down research paradigm in a more egalitarian direction [73] and increase credibility and reduce potential biases by triangulating different data sources [76].

The scoping review showed that participatory approaches in the development of interventions were most often used in school settings and aimed at supporting lifestyle issues or managing illness or disease [27, 33, 34, 36, 39, 40, 45, 47, 48, 50, 51, 54, 56, 60, 62–64]. None of the included studies focusing on school settings reached the highest level of participation and only one study reached level four [63]. It is possible that the school context, with its traditional structure of power, is preserving a top-down approach in the development of school interventions. In addition, researchers, based on their own experience, might be confident in having sufficient knowledge about the school environment and children and young people’s daily lives and how interventions could best be put forward [67]. However, such attitudes can have a negative impact on utilizing the potential of the school as an arena for health interventions that are co-developed in partnership with the children, strategically to prevent the occurrence of ill health and tactically to promote the health of vulnerable groups or individuals with special needs.

In conclusion, researchers often advocate a participatory approach in which children and young people are seen as credible informants on interventions aimed at improving their health and well-being. However, this scoping review has showed a somewhat ambiguous result concerning implementation of this understanding in research practice. In order to recognize children and young people’s capabilities as active contributors instead of passive recipients of researcher-driven interventions, a consistency between the theoretical understanding of a participatory approach and what is undertaken in research practice in developing health interventions together with the target group is needed.

### Strengths and limitations

Despite a focus on a broad research field and a fairly wide range of publication years, only 41 articles met the inclusion criteria. In an effort to provide clarity and focus for the inclusion and exclusion criteria the research group attached great initial importance to discussing concepts linked to the aim, such as “what action is required to be called an intervention” [77, 78]. Furthermore, additional methodological rigour in the inclusion process included two of the authors independently reading the full text articles. If uncertainty occurred related to article selection, the third author also read the article and a final inclusion decision was then reached in consensus [77]. As an aid to transparency and reproducibility a flowchart and additional text from the search process were included in the method section.

Efforts were made to do more than just map and describe the characteristics of included studies by using Shier’s model [21] as an analysis of the levels of participation. The model has been used in enhancing children and young people’s participation in decision-making in society but to our knowledge the model has not been used before in grading of children and young people’s participation levels in the development of interventions in health and well-being. However, we consider the model to be useful in order to map and clearly illustrate at which level children and young people are involved in these research studies and thus pinpoint the state of knowledge.

The search process in our study is, however, subjected to certain limitations since our strategy was restricted to health, social science and educational databases. Although experienced librarians performed extensive literature searches in several research databases resulting in a large number of articles, it is possible that relevant studies were missed. We have selected original research articles, which thus did not include reviews or grey literature that may have contained studies that could contribute understanding to this topic. Despite only original research articles being included in this scoping review, there is a limitation since scoping reviews do not typically include a quality assessment of included studies [77–79]. This review may also have been limited by restricting the search to English articles as it is the most commonly used language in scientific journals [78]. Moreover, we may lack full knowledge of existing international networks in the research area.

A further limitation could be said to exist in the potential risk for the validity of the grading levels of participation. Even though, the study’s research team was multidisciplinary, they all shared the same geographical and academic context. Moreover, based on the variations in journal requirements, it should be acknowledged that these requirements might be lead to publication bias in terms of how the concept of participation was presented.

It was not possible to further discuss country-specific differences in relation to participatory research approaches as the relatively small total of 41 studies were from a total of 13 different countries. Questions regarding differences between countries and contexts in attitudes concerning to what extent children and young people can be, and are involved in the development of interventions, were not part of the aim for this study but are recommended to be included in future research.

## Conclusions

This scoping review showed that work remains in enabling children and young people to influence the development of health interventions. Many studies were keen to discuss the importance of taking children and young people’s views, experiences and suggestions into account when developing interventions. However, relatively few invited these children and young people to share power in the design, implementation and analysis of the research or as partners in the process of developing the intervention. Participatory approaches aiming for a higher level of reciprocity where children and young people work together with the researchers in partnerships are thus warranted. Such studies also need to carefully describe the methods used in the collaboration with children and to use models, such as the one described by Shier, in their description of how and to what extent the children were actually involved. Only then will it be possible to proceed from describing children’s participation from a theoretical point of view to confronting such argumentation based on previous research. Examples and experiences from further research is a necessity for being able to elaborate on what participation actually means and which level of participation that is most appropriate given a certain context or target group. Further research is also needed to investigate to what extent there is a more beneficial outcome from the interventions, depending on whether the children are involved in the development of the intervention or if the intervention is developed solely by the researchers.

## Abbreviations

CINAHL: Cumulative index to nursing and allied health literature
ERIC: Education resources information center
MESH: Medical subject headings
PsycInfo: Psychological information database
UN: United Nations
